# Functional Expression and Characterization of *Schizosaccharomyces pombe* Avt3p as a Vacuolar Amino Acid Exporter in *Saccharomyces cerevisiae*

**DOI:** 10.1371/journal.pone.0130542

**Published:** 2015-06-17

**Authors:** Soracom Chardwiriyapreecha, Kunio Manabe, Tomoko Iwaki, Miyuki Kawano-Kawada, Takayuki Sekito, Siriporn Lunprom, Koichi Akiyama, Kaoru Takegawa, Yoshimi Kakinuma

**Affiliations:** 1 Laboratory of Molecular Physiology and Genetics, Faculty of Agriculture, Ehime University, Matsuyama, Ehime, Japan; 2 Advanced Research Support Center (ADRES), Ehime University, Matsuyama, Ehime, Japan; 3 Laboratory of Applied Microbiology, Faculty of Agriculture, Kyushu University, Fukuoka, Japan; University of Tokyo, JAPAN

## Abstract

In *Saccharomyces cerevisiae*, Avt3p and Avt4p mediate the extrusion of several amino acids from the vacuolar lumen into the cytosol. SpAvt3p of *Schizosaccharomyces pombe*, a homologue of these vacuolar amino acid transporters, has been indicated to be involved in spore formation. In this study, we confirmed that GFP-SpAvt3p localized to the vacuolar membrane in *S*. *pombe*. The amounts of various amino acids increased significantly in the vacuolar pool of *avt3*Δ cells, but decreased in that of *avt3* ^+^-overexpressing *avt3*Δ cells. These results suggest that SpAvt3p participates in the vacuolar compartmentalization of amino acids in *S*. *pombe*. To examine the export activity of SpAvt3p, we expressed the *avt3* ^+^ gene in *S*. *cerevisiae* cells. We found that the heterologously overproduced GFP-SpAvt3p localized to the vacuolar membrane in *S*. *cerevisiae*. Using the vacuolar membrane vesicles isolated from *avt3* ^+^-overexpressing *S*. *cerevisiae* cells, we detected the export activities of alanine and tyrosine in an ATP-dependent manner. These activities were inhibited by the addition of a V-ATPase inhibitor, concanamycin A, thereby suggesting that the activity of SpAvt3p is dependent on a proton electrochemical gradient generated by the action of V-ATPase. In addition, the amounts of various amino acids in the vacuolar pools of *S*. *cerevisiae* cells were decreased by the overproduction of SpAvt3p, which indicated that SpAvt3p was functional in *S*. *cerevisiae* cells. Thus, SpAvt3p is a vacuolar transporter that is involved in the export of amino acids from *S*. *pombe* vacuoles.

## Introduction

Yeast vacuoles are large organelles that function as digestive compartments and also serve as major storage compartments for various amino acids [1–3]. In *Saccharomyces cerevisiae*, the vacuoles contain about 70–90% of the basic amino acids in cells, whereas acidic amino acids are mainly excluded from the organelles, with approximately 90% localized in the cytosol [2]. These differences in the distributions of amino acids suggest the presence of specific transport systems that operate across the vacuolar membrane. It has been reported that 10 amino acids are actively transported into the vacuoles in *S*. *cerevisiae* [4]. These active transport systems are probably co-transporters of H^+^ and amino acids, which are driven by a proton electrochemical gradient generated by the vacuolar type H^+^-ATPase (V-ATPase) [4–6].

Several genes for vacuolar amino acid transporters have been identified and characterized in the budding yeast *S*. *cerevisiae* based on experiments using isolated vacuolar membrane vesicles [7–13]. Two gene families, i.e., AVT and VBA, were found to be involved in vacuolar amino acid transport. In the VBA family, which belongs to the major facilitator superfamily, it has been shown that Vba1p, Vba2p, and Vba3p are involved in the uptake of basic amino acids into vacuoles [8]. In the AVT family, which belongs to the amino acid/auxin permease family, Avt1p is involved in the vacuolar uptake of neutral amino acids and histidine [9,10]. Avt3p and Avt4p are involved in the extrusion of neutral and neutral/basic amino acids from vacuoles, respectively [9,12]. Avt6p is involved in the efflux of acidic amino acids [9,13], and Avt7p is involved in the efflux of several neutral amino acids from vacuoles [11]. Furthermore, other genes that belong to the amino acid/polyamine/choline family and the lysosomal cystine transporter family have been identified as vacuolar amino acid transporters [14,15].

Relatively fewer homologs of the vacuolar amino acid transporters have been found in the genome of the fission yeast *Schizosaccharomyces pombe* compared with *S*. *cerevisiae*, thereby implying less redundancy among the functions of its transporters. Therefore, *S*. *pombe* may be advantageous to understand the physiological roles of vacuolar amino acid transporters. Previously, based on phylogenetic analysis of *S*. *cerevisiae* and *S*. *pombe* genomic database, we found that the genes *fnx1* ^+^, *fnx2* ^+^, *avt5* ^+^, *vba2* ^+^, and *atg22* ^+^ are homologs of the vacuolar amino acid transporters identified in *S*. *cerevisiae*. However, it is quite difficult to characterize the genes of vacuolar transporters in *S*. *pombe* using isolated vacuolar membrane vesicles because the vacuoles are too small in *S*. *pombe* and a procedure has not been established for purifying the vacuolar membrane vesicles from *S*. *pombe* cells. We also found that V-ATPase-dependent vacuolar compartmentalization had a large effect on amino acid uptake by *S*. *pombe* whole cells, so assessing the vacuolar transport activity of amino acids was possible using an indirect assay with whole cells of *S*. *pombe* [16]. Using this whole cell assay, we found that Fnx1p and Fnx2p are involved in the uptake of lysine, asparagine, and isoleucine into vacuoles [16]. In addition, Avt5p is involved in the vacuolar uptake of various amino acids [17]. Vba2p is involved in the uptake of basic amino acids into vacuoles [18], and Atg22p is involved in the uptake of several amino acids into vacuoles, as well as in the maintenance of cellular and vacuolar amino acid pools [19]. In any case, establishing an *in vitro* membrane vesicle system is indispensable for investigating the net transport activities of these transporters.

Under nitrogen starvation, cells utilize the vacuolar amino acid pool as a nitrogen source [20], which is important for maintaining cellular functions [20–22]. Thus, exporters of vacuolar amino acids are expected to be important for recycling amino acids for *de novo* protein synthesis or metabolic pathways [22]. However, the genes of the vacuolar amino acid exporters have not been well characterized in *S*. *pombe*. Recently, Mukaiyama *et al*. indicated that *S*. *pombe* Avt3p (*SPAC3H1*.*09c*, SpAvt3p), a homologue of *S*. *cerevisiae* Avt3p (*YKL146w*) and Avt4p (*YNL101w*), is involved in spore formation by *S*. *pombe* cells [23].

In this study, the *avt3* ^+^ gene of *S*. *pombe* was heterologously expressed in *S*. *cerevisiae* cells, and characterized using isolated vacuolar membrane vesicles. We suggest that SpAvt3p is a vacuolar membrane transporter involved in the extrusion of amino acids from vacuoles.

## Materials and Methods

### Strains, media, and materials

The *S*. *pombe* strains used in this study were the wild-type ARC039 (*h* ^*-*^ *leu1-32 ura4-C190T*) [24], and its gene disruptant, the *avt3*Δ mutant [23]. The *S*. *cerevisiae* strains used in this study were STY3807 (*MATa SUC2 mal mel gal2 CUP1 ura3*Δ::*loxP*) [12] as the wild-type, which was derived from X2180-1B (*MATa SUC2 mal mel gal2 CUP1*) (Yeast genetic stock center, http://www.atcc.org/en/Products/Cells_and_Microorganisms), and its derivatives, the *avt3*Δ*avt4*Δ mutant (STY3828) and the *avt1*Δ*avt3*Δ*avt4*Δ mutant (STY4109) [10,12]. Yeast cells were grown aerobically at 30°C. Standard medium (YES) and synthetic minimal medium (MM) were used for *S*. *pombe*, as described previously [25]. *S*. *cerevisiae* strains were cultured in YPD medium (1% yeast extract, 2% polypeptone, and 2% glucose) or in SD+CA medium (0.17% yeast nitrogen base without amino acids and ammonium sulfate, 0.5% ammonium sulfate, 0.5% casamino acids, 20 mg/L tryptophan, and 2% glucose). *S*. *pombe* cells were transformed using the lithium acetate method [26]. Other manipulations of yeast were preformed according to standard procedures [27,28]. Concanamycin A (CCA) and FM4-64 were purchased from Wako Pure Chemicals Co. (Osaka, Japan) and Invitrogen Corp. (Carlsbad, CA, USA), respectively. l-[^14^C] labeled amino acids were purchased from American Radiolabeled Chemicals Inc. (St Louis, MO, USA), GE Healthcare (Buckinghamshire, UK), and Perkin Elmer Inc. (Waltham, MA, USA).

### Plasmid construction and fluorescence microscopy

To tag the SpAvt3p protein with green fluorescence protein (GFP) at its N-terminus, the open reading frame was amplified by PCR and subcloned into pTN54, a derivative of the thiamine-repressible expression vector pREP41 [29], thereby yielding pTN54-*avt3* ^+^. *S*. *pombe* cells transformed with pTN54-*avt3* ^+^ were grown in MM medium without leucine and thiamine at 30°C for 20 h, and then labeled with FM4-64, a lipophilic dye for vacuolar membrane staining, as described previously [30].

To construct the *avt3* ^+^ expression plasmid for *S*. *cerevisiae*, the GFP-tagged *avt3* ^+^ was subcloned into pRS416GPD [31], thereby obtaining pGPD-GFP-*avt3* ^+^. A single point mutation, *avt3* ^*E469A*^, was constructed using a QuikChange site-directed mutagenesis kit (Stratagene, La Jolla, CA). Staining with FM4-64 in *S*. *cerevisiae* cells was performed as described previously [32]. Cells were observed with an IX71 fluorescence microscope (Olympus, Tokyo, Japan) equipped with a cooled charge-coupled device camera (ImagEMC9100-13; Hamamatsu, Japan). Images were acquired using Metamorph software (Universal imaging, West Chester, PA).

### Transport assays

Cells were cultured in SD+CA medium at 30°C, and then harvested at an OD_660_ of 1.0–1.5. Vacuolar membrane vesicles of *S*. *cerevisiae* were prepared as described previously [8]. To measure the export activity of amino acids, vesicles were preloaded with L-[^14^C]-labeled specific amino acids by incubating for 10 min at 25°C in an assay mixture (500 μL), which comprised 25 mM 2-(*N*-morpholino)ethanosulfonic (MES)- tris(hydroxymethyl)aminomethane (Tris) (pH 7.0), 5 mM MgCl_2_, 25 mM KCl, vesicles (200 μg of protein), and [^14^C]-labeled amino acid (0.1 mM; 4.8–11.1 GBq/mmol). For the CCA treatment, vesicles were incubated with 1 μM CCA for 10 min at 25°C before preloading with [^14^C]-labeled amino acid. The reaction was initiated by adding of 2 mM ATP and then stopped by diluting a 100 μL sample with 5 mL of ice-cold wash buffer (25 mM MES-Tris, pH 7.0, 5 mM MgCl_2_, 25 mM KCl) at specific time points. Vesicles were filtered through cellulose acetate membrane filters (0.45 μm; ADVANTEC, Japan) and washed with 5 mL of ice-cold wash buffer. Radioactivity was measured using a liquid scintillation counter with a xylene scintillator. The uptake activity of amino acids was determined as described previously [8].

### Analysis of amino acids

Ten OD_660_ units of cells were harvested and washed once with 2.5 mM potassium phosphate buffer (pH 6.0) containing 10 mM glucose, and 1.5 M sorbitol (for *S*. *pombe*) or 0.6 M sorbitol (for *S*. *cerevisiae*). Cupric ion treatment was used to prepare vacuolar fractions of *S*. *pombe* and *S*. *cerevisiae* [33,34]. The amino acid contents in the fractions were analyzed with an amino acid analyzer (Hitachi L-8800).

### Immunoblot analysis

Vacuolar membrane vesicles (20 μg of protein) were applied to an SDS-PAGE gel containing 8% polyacrylamide and analyzed by immunoblotting. Anti-GFP serum was purchased from Invitrogen, and anti-vacuolar alkaline phosphatase (Pho8) antibody was obtained from Molecular Probes (Eugene, OR). Immunoblot signals were detected using the ECL system (GE Healthcare). Protein concentrations were determined by the method proposed by Lowry *et al*. [35] with bovine serum albumin as the standard.

## Results

### Intracellular localization of GFP-Avt3p in *S*. *pombe* cells

**Fig 1:**
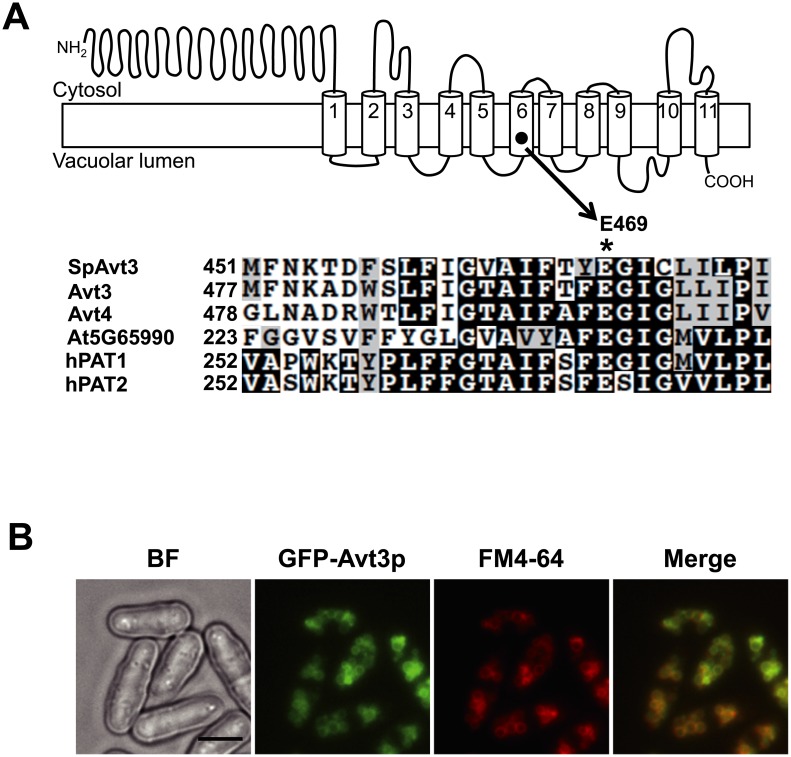
Predicted topology model and intracellular localization of SpAvt3p. (A)*Top*, predicted topology model of SpAvt3p. *Bottom*, sequence alignments of SpAvt3p (Q10074.1) in TM6 (amino acids 451–477) and analogous regions in the homologs according to CLUSTALW: *Saccharomyces cerevisiae* Avt3p and Avt4p (P36062 and P50944, respectively), *Arabidopsis thaliana* At5G65990 (ABH04593), and human hPAT1and hPAT2 (AAI36439 and AAI01104, respectively). Identical and similar residues are denoted by *black boxes* and *gray boxes*, respectively. The conserved glutamate residue is indicated by an asterisk. (B) The *avt3*Δ mutant cells expressing GFP-SpAvt3p fusion protein were subjected to fluorescence microscopy. Vacuolar membranes were stained with FM4-64. BF, bright field; bar, 5 μm.

Recently, we showed that Avt3p (*YKL146w*) and Avt4p (*YNL101w*) are involved in amino acid export from the vacuoles in *S*. *cerevisiae* [12]. Based on phylogenetic relationships with Avt3p and Avt4p, we found that Avt3p in *S*. *pombe* (*SPAC3H1*.*09c*, SpAvt3p) is the closest relative to these proteins in *S*. *cerevisiae* (the shared identities between SpAvt3p with Avt3p and Avt4p were 41% and 34%, respectively). SpAvt3p was predicted to possess 11-transmembrane domains using several programs. Fig 1A shows the topology model of SpAvt3p predicted using the SOSUI program (SOSUI; http://harrier.nagahama-i-bio.ac.jp/sosui/). The alignment of SpAvt3p homologs showed that a glutamate residue at position 469 (E469), which was embedded in the putative sixth transmembrane domain of SpAvt3p, was highly conserved not only in Avt3p and Avt4p in *S*. *cerevisiae*, but also in other eukaryotic homologs (Fig 1A). This conserved glutamate residue was suggested to be essential for the activity of Avt4p in *S*. *cerevisiae* [12].

In addition to its homology with Avt3p and Avt4p, Mukaiyama *et al*. recently indicated that deletion of the *avt3* ^+^ gene impaired sporulation of *S*. *pombe* cells [23], suggesting that SpAvt3p might play an important role in the export of amino acids from vacuoles to the cytosol to maintain cellular functions under starvation conditions. Therefore, we hypothesized that SpAvt3p could be a vacuolar amino acid exporter in *S*. *pombe*.

To investigate the subcellular localization of SpAvt3p in *S*. *pombe* cells, GFP was tagged at the N-terminus of SpAvt3p and expressed in *S*. *pombe avt3*Δ cells. The fluorescence image of GFP-SpAvt3p was merged with the signal of FM4-64, a specific dye for the vacuolar membrane, thereby indicating that SpAvt3p localized exclusively at the vacuolar membrane in *S*. *pombe* (Fig 1B).

### Effects of *avt3* ^+^ expression on the amino acid compositions in vacuoles and the vacuolar morphology of *S*. *pombe*

To assess the involvement of SpAvt3p in the vacuolar extrusion of amino acids, we determined the amino acid contents of the vacuolar pools in *avt3*Δ or *avt3* ^+^-overexpressing *S*. *pombe* cells. The *S*. *pombe* wild-type, *avt3*Δ, and *avt3*Δ/pTN54-*avt3* ^+^ cells were cultured in MM medium without thiamine, and vacuolar amino acid pools were prepared using the cupric ion method [33]. As shown in Table 1, the vacuolar levels of threonine, serine, asparagine, glutamine, glycine, alanine, and proline, as well as basic amino acids (histidine, arginine, and lysine), were obviously increased in the *avt3*Δ cells compared with the wild-type cells. The *avt3*Δ mutation also had moderate effects on the levels of valine and tyrosine, but little effect on those of other amino acids. The increased levels of these amino acids in the vacuolar pool of *avt3*Δ cells were significantly reduced by the overexpression of *avt3* ^+^. These results suggest that SpAvt3p is involved in the efflux of these amino acids from vacuoles.

**Table 1:** Effects of *avt3* ^+^ expression on the amino acid compositions of the vacuolar pools in *S*. *pombe* cells.

Amino acid	Strain			
	Wild-type/pTN54	*avt3*Δ/pTN54 ^(a)^	*avt3*Δ/ pTN54-*avt3* ^+ (b)^	Ratio ^(b/a)^
Asp	39.9 ± 1.8	34.9 ± 1.5	31.9 ± 2.8	0.914
Thr	30.8 ± 2.6	148.1 ± 5.5	27.3 ± 1.5	0.184
Ser	21.3 ± 1.1	71.6 ± 3.1	21.4 ± 2.3	0.299
Asn	6.2 ± 0.3	15.0 ± 0.6	4.6 ± 0.2	0.307
Glu	269.7 ± 30.05	290.7 ± 9.4	280.2 ± 19.0	0.964
Gln	100.1 ± 19.0	316.4 ± 8.0	222.1 ± 13.8	0.702
Gly	21.8 ± 1.7	61.4 ± 2.6	20.4 ± 1.0	0.332
Ala	61.2 ± 8.4	212.0 ± 9.2	49.8 ± 5.8	0.235
Val	24.2 ± 2.0	33.8 ± 3.3	28.7 ± 4.4	0.849
Met	5.3 ± 0.4	5.5 ± 0.3	6.4 ± 0.1	1.163
Ile	11.8 ± 0.7	14.6 ± 0.9	14.1 ± 1.1	0.965
Leu	24.1 ± 0.5	23.8 ± 1.8	25.2 ± 1.5	1.059
Tyr	9.8 ± 0.4	13.2 ± 0.6	10.1 ± 0.3	0.765
Phe	12.4 ± 0.3	13.5 ± 0.7	14.2 ± 0.5	1.052
Trp	3.7 ± 0.2	3.9 ± 0.4	2.7 ± 0.1	0.692
Lys	425.7 ± 25.0	1377.3 ± 51.2	152.4 ± 3.4	0.111
His	258.8 ± 28.7	542.9 ± 25.7	39.4 ± 0.8	0.073
Arg	206.8 ± 7.5	508.3 ± 22.0	71.7 ± 1.8	0.141
Pro	5.1 ± 0.4	13.5 ± 0.4	3.1 ± 0.5	0.230
Total	1538.7 ± 131.1	3700.4 ± 147.2	1025.7 ± 60.9	0.277

The amounts of amino acids (nmol/5 × 10^8^ cells) in the vacuolar fractions of the wild-type, *avt3*Δ, and *avt3* ^+^-overexpressing cells are indicated. The results represent the mean ± SD based on three independent experiments. Cells were grown in MM medium without leucine and thiamine for 20 h, and the vacuolar pools were then prepared as described in the Materials and Methods section. Ratio of vacuolar amino acid amounts in *avt3*Δ/pTN54-*avt3* ^+^ cells (b) to those in *avt3*Δ/pTN54 cells (a) are indicated (b/a).

**Fig 2:**
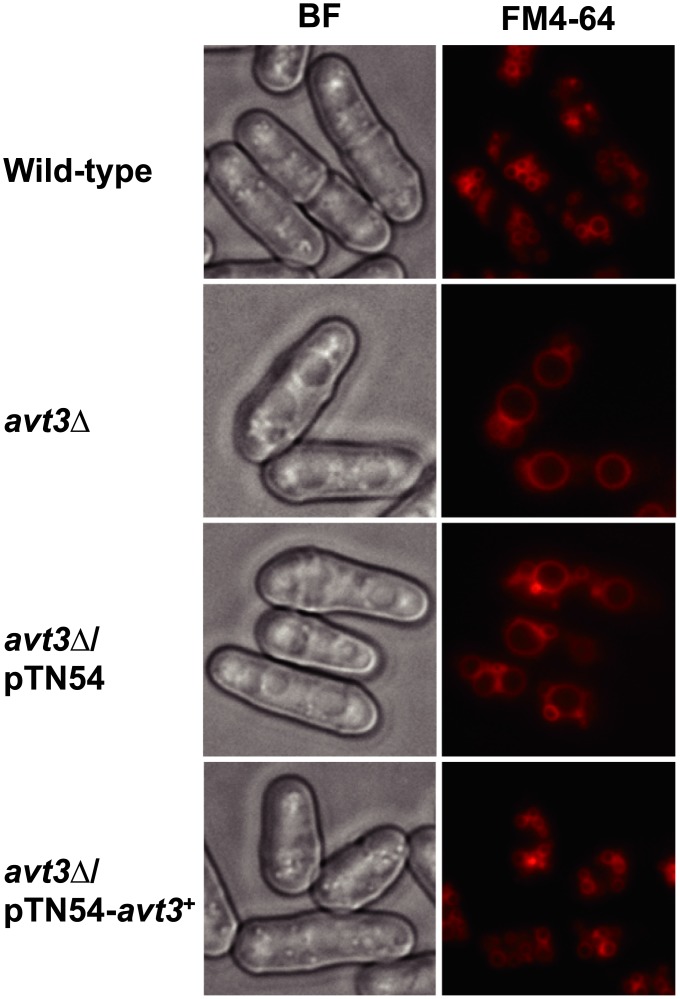
Vacuolar morphology of *S*. *pombe* ARC039 (wild-type), *avt3*Δ, *avt3*Δ/pTN54, and *avt3*Δ/pTN54-*avt3* ^+^. Cells were grown in MM medium without thiamine for 20 h and the vacuolar membranes were then stained with FM4-64. BF, bright field; bar, 5 μm.

Interestingly, we found that the size of vacuoles in *avt3*Δ cells was strikingly increased compared with that in the wild-type cells, and decreased in *avt3* ^+^-overexpressing cells (Fig 2), thereby indicating that the *avt3* ^+^ gene expression also affected the vacuolar morphology.

### SpAvt3p-dependent extrusion of amino acids from *S*. *cerevisiae* vacuolar membrane vesicles

**Fig 3:**
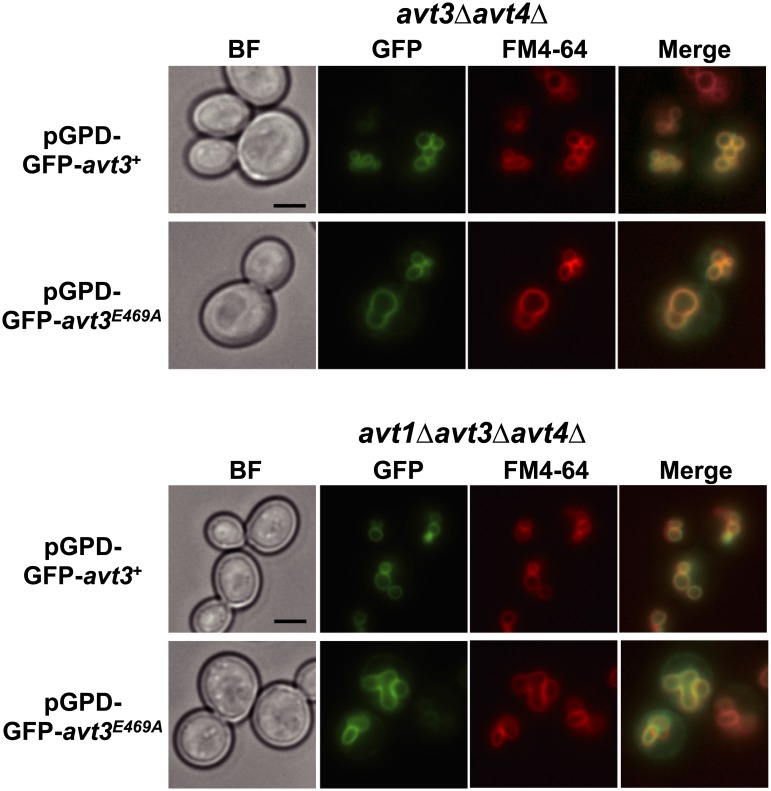
Intracellular localization of SpAvt3p in *S*. *cerevisiae* cells. The *avt3*Δ*avt4*Δ or *avt1*Δ*avt3*Δ*avt4*Δ mutant cells expressing GFP-SpAvt3p or GFP-SpAvt3p^E469A^ fusion protein were subjected to fluorescence microscopy. Vacuolar membranes were labeled with FM4-64. BF, bright field; bar, 3 μm.

Our analysis of the vacuolar amino acid contents indicated that the overproduction of SpAvt3p decreased the levels of various neutral and basic amino acids in the vacuolar pools in *S*. *pombe* cells (Table 1). Further, we examined whether SpAvt3p is involved in the vacuolar export of amino acids by using isolated vacuolar membrane vesicles. Many vacuolar transporters have been characterized using isolated vacuolar membrane vesicles in *S*. *cerevisiae* [8–12]. However, the isolation of vacuolar membrane vesicles is difficult in *S*. *pombe* because a standard method has not been established for isolating vacuoles due to their small size. Thus, SpAvt3p was heterologously overproduced in *S*. *cerevisiae* cells to examine its transport activity. To reduce the background activities of efflux and influx of amino acids by vacuolar membrane vesicles, either the *avt3*Δ*avt4*Δ double mutant [12] or the *avt1*Δ*avt3*Δ*avt4*Δ triple mutant [10] of *S*. *cerevisiae* was used as the host strain. Both GFP-SpAvt3p and GFP-SpAvt3p^E496A^ localized exclusively at the vacuolar membrane in *avt3*Δ*avt4*Δ and *avt1*Δ*avt3*Δ*avt4*Δ cells (Fig 3), and the cellular levels of these proteins in the total cell lysates were almost equal (data not shown).

**Fig 4:**
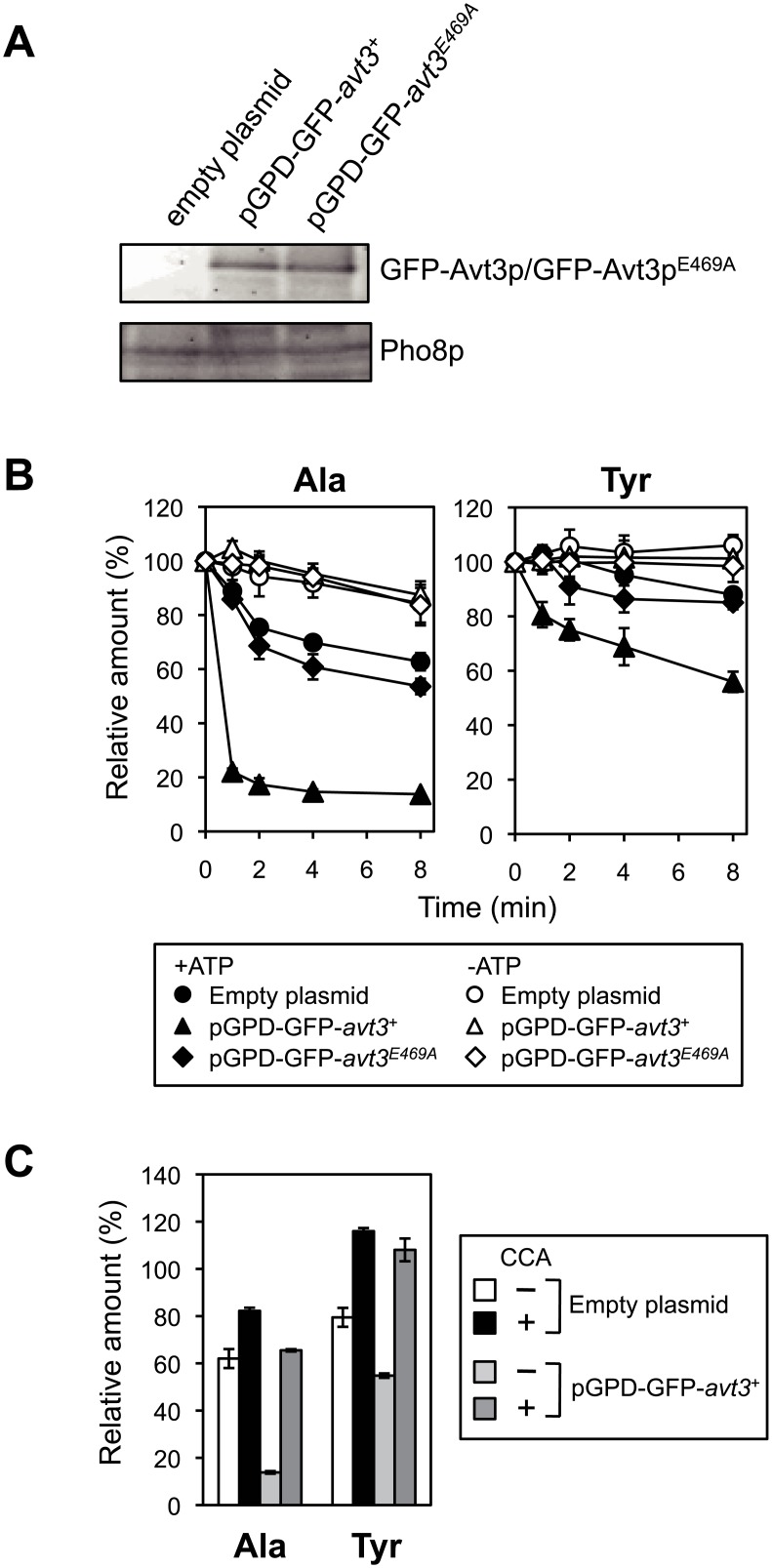
SpAvt3p-dependent extrusion of amino acids by vacuolar membrane vesicles. (A) Immunoblot analysis of GFP-SpAvt3p and GFP-SpAvt3p^E469A^ in the vacuolar membrane vesicles isolated from *S*. *cerevisiae avt1*Δ*avt3*Δ*avt4*Δ mutant cells. Vacuolar membrane vesicles were prepared and analyzed by immunoblotting using anti-GFP serum and anti-Pho8 antibody. Pho8p was detected as the loading control. (B) Alanine and tyrosine export by vacuolar membrane vesicles. [^14^C]-labeled amino acids were preloaded into the vacuolar membrane vesicles isolated from *avt1*Δ*avt3*Δ*avt4*Δ cells carrying an empty plasmid (*circles*), pGPD-GFP-*avt3* ^+^ (*triangles*), or pGPD-GFP-*avt3* ^*E469A*^ (*diamonds*). The export assay was performed in the presence (*black symbols*) or absence (*white symbols*) of 2 mM ATP. Preloaded vesicles were removed immediately before (0 min) or at 1, 2, 4, and 8 min after the addition of ATP, and collected on cellulose acetate membrane filters. The amount of preloaded [^14^C]-labeled amino acids at 0 min was taken as 100%. The relative amounts trapped on the filters are shown. The values represent the mean ± SD based on at least three independent experiments. (C) Effects of CCA on ATP-driven alanine and tyrosine export. The experiments were performed as described above. Vacuolar membrane vesicles were incubated with 1 μM CCA for 10 min before loading with [^14^C]-labeled amino acids. The amount of preloaded [^14^C]-labeled amino acids at 0 min was taken as 100%. The relative amounts trapped on the filters at 8 min after the addition of ATP are shown. The values represent the mean ± SD based on at least three independent experiments: *avt1*Δ*avt3*Δ*avt4*Δ cells carrying an empty plasmid without (*white bar*) or with (*black bar*) CCA, and pGPD-GFP-*avt3* ^+^ without (*light gray bar*) or with CCA (*dark gray bar*).

To examine the export activity of amino acids, the vacuolar membrane vesicles were isolated from *avt1*Δ*avt3*Δ*avt4*Δ cells that overproduced SpAvt3p. The expression levels of GFP-SpAvt3p and GFP-SpAvt3p^E469A^ in the vacuolar membrane vesicles were almost equal (Fig 4A). [^14^C]-labeled alanine or tyrosine was preloaded into vacuolar membrane vesicles for 10 min, and the amounts of these [^14^C]-labeled amino acids remaining in the vesicles after the addition of 2 mM ATP were determined. The efflux of these amino acids from the vesicles was slight in the absence of ATP. The amount of alanine, as well as tyrosine, remaining in the *avt1*Δ*avt3*Δ*avt4*Δ vesicles was reduced partially by the addition of ATP (Fig 4B, black circles). In agreement with the data of amino acid composition (Table 1), the ATP-dependent export of alanine was enhanced greatly by the overproduction of SpAvt3p and that of tyrosine was enhanced moderately (Fig 4B, black triangles). The overproduction of SpAvt3p^E469A^ did not affect the activity (Fig 4B, black diamonds). As shown in Fig 4C, these export activities were inhibited by the addition of CCA, a specific inhibitor of V-ATPase, suggesting that the activity of SpAvt3p depends on a proton electrochemical gradient generated by the action of V-ATPase.

### Effects of *avt3* ^+^ expression on the uptake activity of basic amino acids by *S*. *cerevisiae* vacuolar membrane vesicles

**Fig 5:**
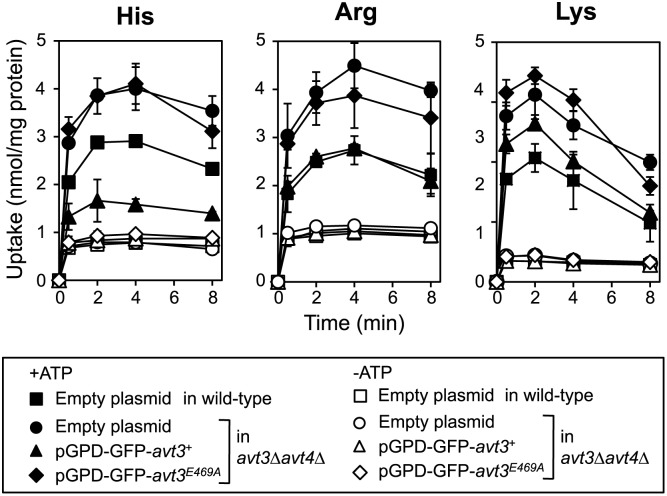
Effects of *avt3* ^+^ expression on the ATP-dependent uptake of basic amino acids by vacuolar membrane vesicles. Vacuolar membrane vesicles were isolated from the wild-type cells carrying an empty plasmid (*squares*), the *avt3*Δ*avt4*Δ cells carrying an empty plasmid (*circles*), pGPD-GFP-*avt3* ^+^ (*triangles*), and pGPD-GFP-*avt3* ^*E469A*^ (*diamonds*). The amino acid uptake assay was performed with (*black symbols*) or without (*white symbols*) 2 mM ATP. The values represent the mean ± SD based on at least three independent experiments.

We examined whether the overproduction of SpAvt3p affected the transport activity of basic amino acids using isolated vacuolar membrane vesicles. It is difficult to detect the export activity of basic amino acids, which are imported naturally rather than exported from vacuoles, so we examined the effect of SpAvt3p overproduction on the net uptake of basic amino acids using vacuolar membrane vesicles without preloading. The vacuolar membrane vesicles were isolated from the wild-type or the *avt3*Δ*avt4*Δ cells carrying an empty plasmid, pGPD-GFP-*avt3* ^+^, or pGPD-GFP-*avt3* ^*E469A*^. As shown in Fig 5, the ATP-dependent uptake of basic amino acids was higher in the vesicles from the *avt3*Δ*avt4*Δ cells compared with those from the wild-type cells, indicating that the disruption of *AVT3* and *AVT4* blocked the efflux of basic amino acids from vacuoles. The overproduction of SpAvt3p significantly decreased the uptake activities (Fig 5, black triangles), whereas the overproduction of SpAvt3p^E469A^ had no effect (Fig 5, black diamonds). These results suggest that SpAvt3p mediates the export of basic amino acids from vacuoles, as well as neutral ones.

### Vacuolar amino acid composition of *S*. *cerevisiae* cells overexpressing *avt3* ^+^ or *avt3* ^*E469A*^

**Fig 6:**
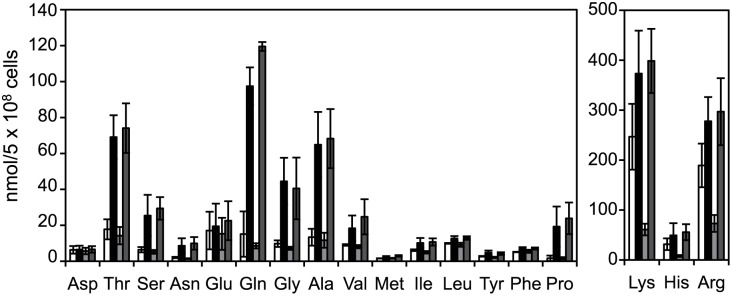
Effects of *avt3* ^+^ expression on the vacuolar amino acid composition of *S*. *cerevisiae*. The vacuolar pools of *S*. *cerevisiae* were prepared and analyzed using an amino acid analyzer. The results represent the mean ± SD based on at least three independent experiments: wild-type cells carrying an empty plasmid (*white bar*), *avt3*Δ*avt4*Δ cells carrying an empty plasmid (*black bar*), pGPD-GFP-*avt3* ^+^ (*light gray bar*), and pGPD-GFP-*avt3* ^*E469A*^ (*dark gray bar*).

The results of *in vitro* experiments using isolated vacuolar membrane vesicles indicated that SpAvt3p is involved in the export of neutral and basic amino acids from vacuoles (Figs 4 and 5). Further, we examined the effect of *avt3* ^+^ gene overexpression on vacuolar amino acid compartmentalization in *S*. *cerevisiae* cells. The neutral and basic amino acids, but not the acidic ones, were highly accumulated in the vacuolar pool of *avt3*Δ*avt4*Δ cells compared with that of the wild-type cells (Fig 6) [12]. In agreement with the results of the transport assay (Figs 4 and 5), the increase in the vacuolar levels of these amino acids were decreased by overexpressing *avt3* ^+^ in *avt3*Δ*avt4*Δ cells, which suggests that SpAvt3p was functional in the vacuolar compartmentalization of amino acids in *S*. *cerevisiae* cells. By contrast, the overexpression of *avt3* ^*E469A*^ had no effect on the vacuolar levels of neutral and basic amino acids in *avt3*Δ*avt4*Δ cells. The levels of GFP-SpAvt3p and GFP-SpAvt3p^E469A^ levels were almost equal according to western blotting with anti-GFP serum (data not shown), which indicates that the difference in their activities was not caused by unequal amounts of the overproduced proteins. These results suggest that the SpAvt3p transporter is involved in the export of various amino acids from vacuoles in *S*. *pombe* cells.

## Discussion

In this study, we obtained evidence that SpAvt3p localizes to the vacuolar membrane (Fig 1) where it plays a crucial role in the compartmentalization of amino acids in the vacuoles of *S*. *pombe* cells (Table 1). The overproduction of SpAvt3p in *S*. *cerevisiae* cells enhanced the efflux of alanine and tyrosine and reduced the uptake of basic amino acids by vacuolar membrane vesicles (Figs 4 and 5). The contents of amino acids in *S*. *cerevisiae* vacuoles were reduced by the overproduction of SpAvt3p (Fig 6). These results suggest that the SpAvt3p mediates the export of various amino acids from the vacuoles of *S*. *pombe*.

The ATP-dependent export of alanine and tyrosine by vacuolar membrane vesicles was accelerated by the overproduction of SpAvt3p. Alanine was totally extruded from the vesicles isolated from the SpAvt3p-overproducing cells, whereas tyrosine still remained in the vesicles (Fig 4B). The residual amount of tyrosine in vesicles might be due to transport systems related to the ATP-dependent uptake of tyrosine. The ATP-dependent uptake activity of basic amino acids into the vesicles was impaired by the overexpression of *avt3* ^+^ (Fig 5), which suggests that SpAvt3p is also involved in the export of basic amino acids. However, a method has not been established for detecting the ATP-dependent export activity of basic amino acids. In the transport assay, we used purified vacuolar membrane vesicles from the *vba1*Δ*vba2*Δ*vba3*Δ strain, in which the genes involved in the uptake of basic amino acids into vacuoles were disrupted [8], but basic amino acids were still highly accumulated in the vesicles after the addition of ATP. We hypothesize that unknown vacuolar transporters are responsible for the uptake of basic amino acids. It should be noted that Ypq1p, a member of the transporter-opsin-G protein-coupled receptor superfamily, has been suggested to be involved in the vacuolar uptake of lysine and arginine [36]. Analyses of the export activity using the cells, in which the genes for Ypq1p and its homologs were disrupted, are currently in progress.

We suggest that SpAvt3p is a vacuolar exporter for basic and neutral amino acids; however, the phylogenetic analysis indicated that SpAvt3p shared higher similarity with Avt3p rather than Avt4p in *S*. *cerevisiae*. Avt3p and Avt4p are involved in the extrusion of neutral and neutral/basic amino acids from vacuoles, respectively [12]. After comparing the amino acid sequences of SpAvt3p, Avt3p, and Avt4p, we could not find any characteristic residue(s) that might determine the specificity for amino acids. Thus, the binding sites for the substrates of these amino acid exporters should be investigated further.

In normal growth conditions, *S*. *pombe* cells contain many small vacuoles. The vacuoles fuse rapidly when cells are suspended in water in response to hypotonic stress. By contrast, vacuole fission can occur when cells are under hypertonic stress [1,37], thereby suggesting that changes in the vacuolar size and compartment number may correspond to the uptake or release of water and ions by vacuoles. Furthermore, the lipid composition, proton homeostasis, and regulatory proteins are also important for vacuolar morphology [38–40]. We found that the vacuoles were larger in *avt3*Δ cells (Fig 2). It has been reported that the deletion of *btn1* ^+^, a homolog of the human Batten disease gene *CLN3*, results in enlarged and more alkaline vacuoles [41]. Interestingly, Btn1p is a vacuolar protein that appears to maintain the balance of cellular basic amino acid levels in yeast [42]. We postulate that the unusual levels of vacuolar amino acids in *avt3*Δ cells might have affected the vacuolar morphology. However, further experiments are needed to determine how SpAvt3p is involved in the vacuolar morphology.

During nitrogen starvation, the sporulation of yeast cells is impaired by *atg* mutations, which cause a defect in autophagy, a bulk protein degradation process that generates amino acids in vacuoles under nitrogen starvation [22,23]. Mukaiyama *et al*. reported that defects in the sporulation of fission yeast was caused by the insufficient expression of proteins due to a lack of amino acids in the cytosol, which suggests that vacuolar amino acid exporters are important during nitrogen starvation [23]. Interestingly, it has been reported that the *avt3*Δ mutant cells exhibit a defect in sporulation [23]. Our studies showed that amino acids were largely accumulated in the vacuolar pool of *avt3*Δ mutant in nutrient-rich condition (Table 1). Thus, we propose that a vacuolar amino acid exporter, SpAvt3p, may play an important role in recycling amino acids from vacuoles into the cytosol during nitrogen starvation in *S*. *pombe* cells. In addition, the predicted topology of SpAvt3p shows that the protein has a long hydrophilic region at its N-terminus (Fig 1A). Surprisingly, we found that deleting the N-terminal region of SpAvt3p caused enlargement of the vacuoles (unpublished data), suggesting that the N-terminal region of SpAvt3p might be involved in its activity. Currently, we are studying the effects of the N-terminal region of SpAvt3p on spore formation and vacuolar amino acid compartmentalization under nitrogen starvation.

In this study, we successfully achieved the heterologous expression of *S*. *pombe avt3* ^+^ in the vacuolar membrane of *S*. *cerevisiae* cells and characterized SpAvt3p as a vacuolar amino acid exporter. Previously, we determined the amino acid uptake activity of *S*. *pombe* Vba2p using isolated vacuolar membrane vesicles from *vba2* ^+^-overexpressing *S*. *cerevisiae* cells [43]. However, GFP-Atg22p and GFP-Avt5p did not localize appropriately to the vacuolar membrane in *S*. *cerevisiae* cells (unpublished data). Thus, the sorting signals of vacuolar membrane proteins require further elucidation.
